# A Disturbance Compensation Control Strategy for Rotational Speed Standard Device Based on AMB System

**DOI:** 10.3390/s24103093

**Published:** 2024-05-13

**Authors:** Yulin Chen, Lei Du, Qiao Sun, Jie Bai

**Affiliations:** Division of Mechanics and Acoustics Metrology, National Institute of Metrology, Beijing 100029, China; chenyl@nim.ac.cn (Y.C.); sunq@nim.ac.cn (Q.S.); baijie@nim.ac.cn (J.B.)

**Keywords:** rotational speed standard device, AMB, force compensation control

## Abstract

The rotational speed standard device that can carry loads is the key device for calibrating passive rotational speed sensors. The rotor of the passive rotational speed sensor is connected to the rotor of the standard speed device through a coupling, and the standard reference speed is provided by the standard device. Due to the rotor eccentricity, the unbalanced force of the rotor occurs, and it can not only affect the rotational speed accuracy but can also damage the mechanical bearings of the standard speed device. To solve this issue, a method for suppressing the unbalanced force of the speed standard device based on an active magnetic bearing (AMB) force compensation system is proposed. First, the overall structure of the system is briefly introduced. Then, the force feedback control system model with the AMB as the force actuator is established, and a PI controller is designed to achieve the disturbed force control. Finally, a semi-physical simulation experimental platform is built to verify the effectiveness of the proposed method. The experimental results show that the AMB force compensation system can reduce 84.4%, 81.6%, and 79.8% of the unbalanced vibration force at the frequency of 30 Hz, 90 Hz, and 150 Hz, respectively.

## 1. Introduction

The high-precision rotational speed standard device can provide rotational speed traceability for rotating equipment and is the key equipment for achieving the rotational speed calibration of the rotating equipment [1,2]. Rotating equipment is widely used in many advanced equipment in different fields, such as wind turbines, aircraft engines, precision machine tools, intelligent cars, etc. The accurate, effective, and reliable measurement of the rotational speed is the basis for the internal control systems to achieve high-precision rotational speed control, and is also a guarantee for rotating equipment to realize their core functions [3,4,5]. Therefore, the calibration of the speed measurement sensors plays an essential role in its safe and reliable operation. Rotating equipment can be divided into two types, active and passive. The rotor of the active rotating equipment can actively rotate, relying on its own speed control system, so its rotational speed sensor can be calibrated using a rotational speed frequency meter. As for the passive rotating equipment, due to the lack of an internal speed control system, it has to rotate passively, relying on a rotational speed standard device, thereby achieving the rotational speed generation and calibration. Therefore, a high-precision speed standard device with load capacity is necessary for the rotational speed calibration of passive rotating equipment, and it is the main research object of this article.

**Figure 1:**
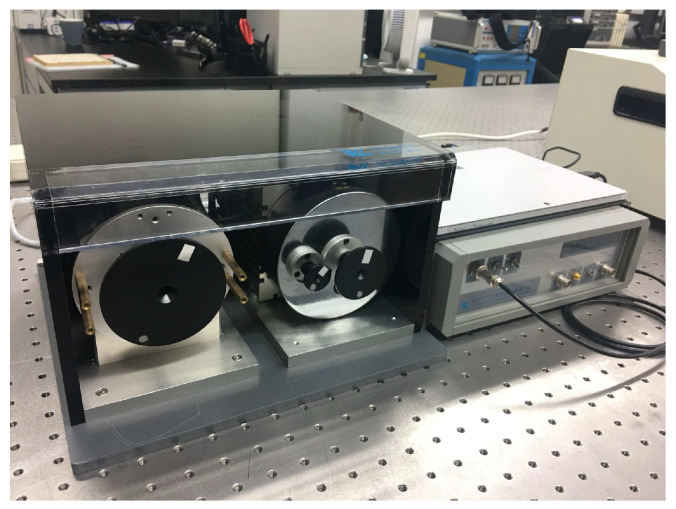
Traditional rotational speed standard device.

The rotational speed standard device is essentially a motor with a turntable or a coupling, but it has a wide speed range and a high rotational speed accuracy. The traditional rotational speed standard device is shown in Figure 1, and some other types of rotational speed standard devices are shown in reference [6].

The study of the rotational speed standard device started early. In the year 1980, the National Institute of Metrology, China (NIM) developed a rotational speed standard device, which had a speed range of 30–100,000 r/min with a measurement uncertainty of 1 × 10^−4^ (*k* = 3). Subsequently, relevant standard devices have also been established in some other countries, such as Singapore, South Korea, Thailand, etc. And the rotational speed standard device was developed with a speed range of 10–100,000 r/min and the measurement uncertainty was 1 × 10^−5^ (*k* = 3) [6,7]. The above high-precision rotational speed standard devices all work under conditions of no load or extremely low load and can be used to calibrate different types of tachometers, speed measuring instruments, and speed sensors. However, some new requirements for the rotational speed calibration have occurred. Some passive rotating equipment, such as a car’s wheel speed sensor calibration device, require a rotational speed standard device to drive their shaft, generating speed and complete calibration. The eccentricity of the load rotor and the rotor of the rotational speed standard device will generate significant unbalanced vibration force, which not only affects the precision of the standard rotational speed but can also damage the internal mechanical bearings of the high-precision rotational speed standard device, affecting the service life of the device. Therefore, the main research objective of this article is to suppress the rotor unbalanced force.

It is well known that the main disturbance force comes from the axis deviation between the rotor of the standard device and the loaded rotor. The frequency and amplitude of the unbalanced force will rise with the increase in rotational rotor speed. The problem of the unbalanced vibration is widely presented in many high-speed rotor systems, such as blowers, compressors, molecular pumps, and torque gyroscopes [8,9,10]. At present, magnetically suspended rotor technology has been applied to these devices to achieve micro-vibration and high rotational speed. Active magnetic bearings (AMBs) are used to replace the mechanical bearings, and the rotor is suspended by the AMB rotor control system. Then, many active vibration suppression algorithms are developed to reduce vibration forces or rotor vibration displacement. Chen Qi et al. proposed a method to suppress the synchronous vibration current of the AMB by combining a notch filter in parallel with the displacement feedback controller. They proved that a notch filter in parallel with the displacement feedback controller has the advantages of faster convergence speed and deeper notch depth. When the synchronous frequency control current is filtered out, the AMB will not provide the synchronous frequency control force, and the rotor will rotate around the inertia axis to reduce the unbalanced vibration. In addition, they also proposed a synchronous unbalanced vibration suppressing method without introducing the rotational rotor speed signal [11,12]. Cui Peiling et al. proposed an active vibration control algorithm based on a multi-synchronous rotational coordinate transformation method. It can use a controller to simultaneously suppress vibration forces in two directions, which has the advantages of saving computational resources, simple operation, and faster response speed [13]. Du Liang et al. proposed a high-precision closed-loop detection method for synchronous signals based on synchronous rotating frame transform, which suppresses the synchronous current by detecting and compensating for synchronous components in the output signal of the displacement sensors [14]. Liu Chao et al. proposed an automatic balance control strategy using the sliding mode observer and the adaptive compensation method to overcome the effects of parameter perturbations and improve the accuracy of automatic balance control in magnetic bearing rotor systems [15]. The high-speed rotor system based on magnetic levitation technology can avoid the damage to the stator caused by unbalanced vibration, but this method also has some problems. Due to the strong gyroscopic effects and the first-order mode transition problem of the rotor, the control algorithm of the magnetic levitation rotor system will be very complex. The magnetically suspended rotor system will face the risk of instability, and once the rotor becomes unstable, it will cause irreversible damage to the system.

In this paper, focusing on the problem of unbalanced force suppression of the rotational speed standard device, a disturbance force compensation control strategy based on an AMB force compensation system is proposed. From the perspective of the development of the rotational speed standard device, the suppression method of the unbalanced vibration force based on the AMB force compensation system is an innovative study. In addition, the AMB system is not used to suspend the rotor but serves as a force compensation system. This method not only has a simple algorithm but also can reduce the unbalanced force without changing the main structure of the traditional speed standard device; regarding this aspect, it also has some innovation.

## 2. Modeling and AMB Force Compensation Method

Firstly, the structure of the rotational speed standard device with an AMB force compensation system is introduced.

### 2.1. Structure of the Rotational Speed Standard Device with an AMB Force Compensation System

**Figure 2:**
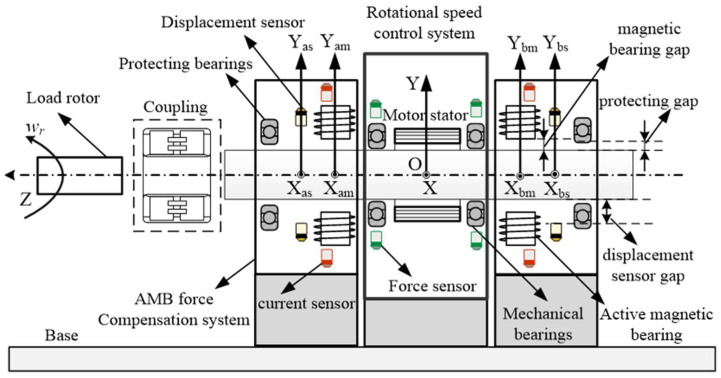
Structure of the rotational speed standard device with AMB force compensation system.

The rotational speed standard device mainly consists of a rotational speed control system and an AMB force compensation control system, as shown in Figure 2.

The load rotor of the passive speed measurement device is connected to the rotational speed standard device through a coupling, and then the standard device provides the standard rotational speed to complete the rotational speed calibration. The rotational speed control system has the same structure as the conventional rotational speed standard device, and it is mainly composed of a motor control system and two mechanical bearings, but integrates many force sensors.

As for the AMB force compensation system, it is the main research object of this article, so it will be introduced in detail. It can be seen from Figure 2 that two AMBs are added to both sides of the rotational speed standard device. The AMB force compensation system mainly includes two radial AMBs, two radial displacement sensors, two protecting bearings, and a common controller. The rotor of the rotational speed standard device is still supported by two mechanical bearings inside the rotational speed control system. The AMB force compensation control system generates appropriate compensation force based on the force sensor signal to reduce the force acting on the mechanical bearings. In general, the clearance between the inner and outer rings of mechanical bearings is much smaller than the magnetic bearing gap, so the rotor will not touch the magnetic bearing. However, when there are deviations in the assembly process, there still remains a risk of collision, so protecting bearings are necessary. Two protecting bearings inside the AMB force compensation system can protect the magnetic bearing and the displacement sensor from the contact of the rotor. There is a submillimeter level protecting gap between the protecting bearings and the rotor, which is smaller than that of the magnetic bearing gap and the displacement sensor gap.

The displacement sensor in the AMB force compensation control system is currently not used in this article, but the authors still display it in Figure 2. The authors want to express that, when the mechanical bearings inside the rotational speed control system are replaced with two protecting bearings with a submillimeter level gap, the rotor can be suspended based on displacement control. Further researches can be conducted on the speed standard device based on the newly established magnetic suspended rotor system.

### 2.2. Force Analysis

**Figure 3:**
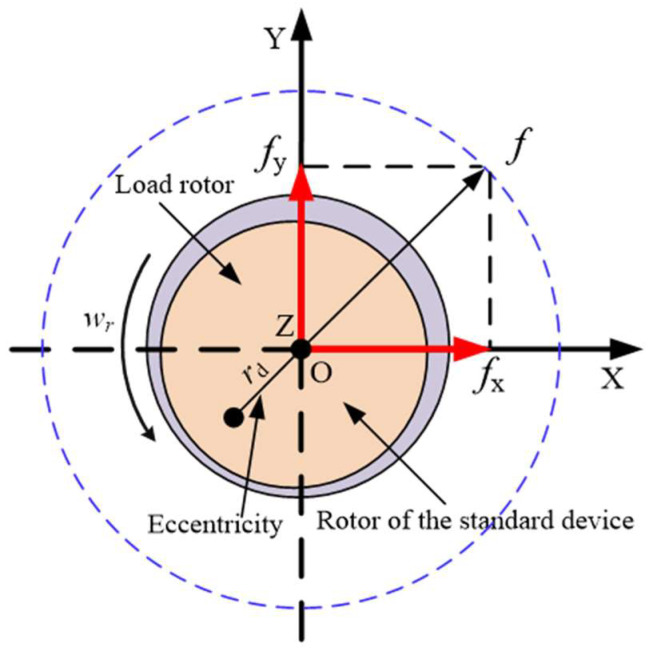
Schematic diagram of rotor unbalanced vibration.

**Figure 4:**
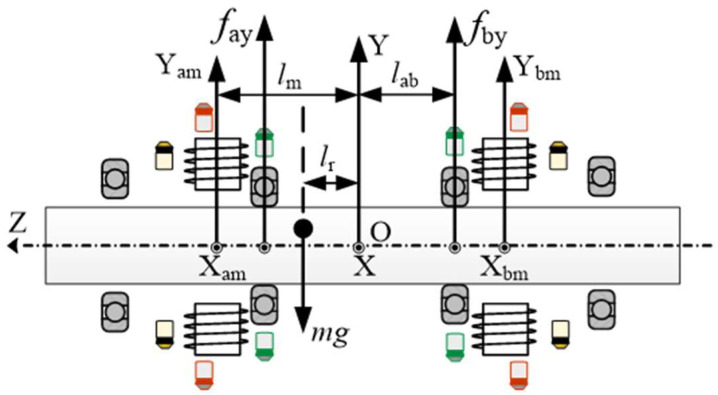
Schematic diagram of system structural parameters.

In order to concisely describe the disturbance suppression method, the force situation of the rotor is first analyzed. The basic structures of the unbalanced force and AMB force compensation system are shown in Figure 3 and Figure 4.

**Figure 5:**
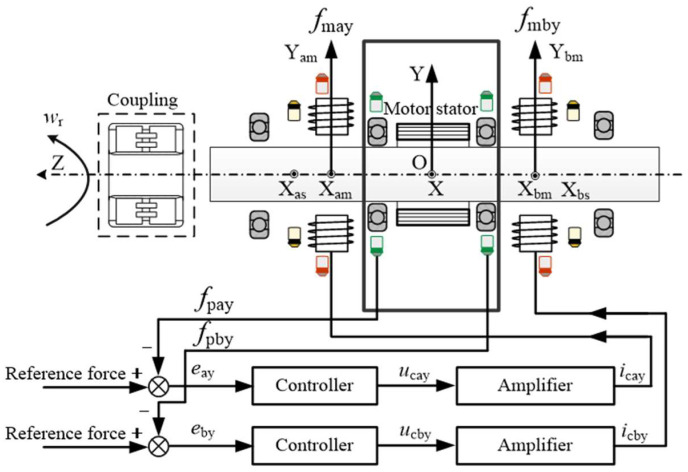
Structure of the rotational speed standard device with the AMB force compensation system.

In Figure 3 and Figure 4, *r_d_* is the distance between the center of mass of the rotor and the geometric center after adding the load on the rotor; *l_r_* is the displacement of the center of mass deviating from the geometric center in the Z direction. The coordinate systems are also shown in these two figures; the vertical upward direction is the Y direction and the horizontal outward direction is the X direction. The actual imbalance of the rotor is more complex but, due to the presence of force sensors, it is not necessary to establish a complex vibration force model. When the rotor rotates at angular velocity *w_r_*, it is stably supported on two mechanical bearings. The equivalent unbalanced disturbing forces in the X and Y directions are defined as

(1) $$\left\{\begin{array}{c} f_{udx}=mw_{r}^{2}r_{d}\cos(w_{r}t+\varphi_{0})\\ f_{udy}=mw_{r}^{2}r_{d}\sin(w_{r}t+\varphi_{0}) \end{array}\right.$$

where *φ*_0_ is the initial angle when the center of mass deviates from the X direction at the initial position. Due to the small clearance between the inner and outer rings of mechanical bearings, the radial motion of the rotor can be ignored. If the force compensation system is not activated, the force acting on the rotor can be described as

(2) $$\left\{\begin{array}{c} mw_{r}^{2}r_{d}\sin(w_{r}t+\varphi_{0})-mg=-(f_{ay}+f_{by})\\ mw_{r}^{2}r_{d}\cos(w_{r}t+\varphi_{0})=-(f_{ax}+f_{bx})\\ -\left[mw_{r}^{2}r_{d}\sin(w_{r}t+\varphi_{0})-mg\right]l_{r}=-(f_{by}-f_{ay})l_{ab}\\ mw_{r}^{2}r_{d}\cos(w_{r}t+\varphi_{0})l_{r}=-(f_{ax}-f_{bx})l_{ab} \end{array}\right.$$

where *m* is the mass of the rotor, g is the gravitational acceleration, *f_ax_* and *f_bx_* are the forces supported by mechanical bearings A and B in the X direction, *f_ay_* and *f_by_* are the forces supported by mechanical bearings A and B in the Y direction; *l_ab_* is the distance between the mechanical bearings and the origin O. Equation (2) can be further described as

(3) $$\left\{\begin{array}{c} -\frac{1}{2}\left(1+\frac{l_{r}}{l_{ab}}\right)mw_{r}^{2}r_{d}\cos(w_{r}t+\varphi_{0})=f_{ax}\\ -\frac{1}{2}\left(1-\frac{l_{r}}{l_{ab}}\right)mw_{r}^{2}r_{d}\cos(w_{r}t+\varphi_{0})=f_{bx}\\ -\frac{1}{2}\left(1+\frac{l_{r}}{l_{ab}}\right)\left(mw_{r}^{2}r_{d}\sin(w_{r}t+\varphi_{0})-mg\right)=f_{ay}\\ -\frac{1}{2}\left(1-\frac{l_{r}}{l_{ab}}\right)\left(mw_{r}^{2}r_{d}\sin(w_{r}t+\varphi_{0})-mg\right)=f_{by} \end{array}\right.$$

when the AMB force compensation system is activated, the basic working principle is shown in Figure 5.

Firstly, two AMBs are located on both sides of the rotor. The force sensor detects the force acting on two mechanical bearings *f_p_*, and it is transmitted to the controller after signal conditioning. The force feedback controller calculates the corresponding control quantity based on the control algorithm, and then the corresponding control currents of the magnetic bearing coils are generated through the power amplifier (AMP) module. The output control force of the magnetic bearing reduces the force acting on the mechanical bearing. When the closed-loop system reaches the steady state, the force acting on the mechanical bearings tends to approach the reference force, and the reference force is set to zero in general.

The AMBs are the force actuators of the force compensation system, and they can provide electromagnetic force to the rotor without contact. The electromagnetic bearings and permanent magnet biased hybrid magnetic bearings are the main forms of the magnetic bearings. Since the current stiffness and displacement stiffness of electromagnetic bearings can be adjusted by adjusting the bias current, this system adopts electromagnetic bearings. The force model of the electromagnetic bearing has been extensively derived in the literature, so this article will not provide a detailed introduction for it. It can be described as

(4) $$f_{m}(i,\delta_{m})=k_{b}[\frac{{\left(I_{0}+i\right)}^{2}}{{\left(\delta_{0}-\delta_{m}\right)}^{2}}-\frac{{\left(I_{0}-i\right)}^{2}}{{\left(\delta_{0}+\delta_{m}\right)}^{2}}]$$

where *k_b_* is the coefficient related to the vacuum permeability, number of turns of magnetic bearing coils, and cross-sectional area of magnetic bearings; *I*_0_ is the bias current and *i* is the control current; *δ*_0_ is the unilateral magnetic gap and *δ_m_* is the displacement of the rotor away from the magnetic center of the magnetic bearings. In order to facilitate the controller design, the above force model of magnetic bearings can be further linearized as

(5) $$f_{m\lambda }=k_{i}i_{\lambda }+k_{h}\delta_{m\lambda },\lambda \in ax,bx,ay,by$$

where *k_i_* and *k_h_* are the current stiffness and displacement stiffness, respectively [16], which are the constant coefficients related to *k_b_*, bias current *I*_0_, and unilateral magnetic gap *δ*_0_. Since *k_b_* and *δ*_0_ are generally determined after the designing and processing of the magnetic bearing, *k_i_* and *k_h_* can be adjusted by changing the bias current *I*_0_. In addition, before designing the controller, an appropriate value of *I*_0_ is selected and will not change unless the controller is going to be designed again. At this time, the current stiffness and displacement stiffness are also determined. It can be seen that the magnetic bearing force is linear with the control current *i* and the rotor displacement *δ_m_*.

When the force compensation system is activated

(6) $$\left\{\begin{array}{c} mw_{r}^{2}r_{d}\sin(w_{r}t+\varphi_{0})-mg+(f_{ay}+f_{by})+(f_{may}+f_{mby})=0\\ mw_{r}^{2}r_{d}\cos(w_{r}t+\varphi_{0})+(f_{ax}+f_{bx})+(f_{max}+f_{mbx})=0\\ -\left[mw_{r}^{2}r_{d}\sin(w_{r}t+\varphi_{0})-mg\right]l_{r}+(f_{by}-f_{ay})l_{ab}+(f_{mby}-f_{may})l_{m}=0\\ mw_{r}^{2}r_{d}\cos(w_{r}t+\varphi_{0})l_{r}+(f_{ax}-f_{bx})l_{ab}+(f_{max}-f_{mbx})l_{m}=0 \end{array}\right.$$

Since there is a short distance between the mechanical bearing and the magnetic bearing, *l_m_* ≈ *l_ab_*. Combining (6) and (5), the force situation of the rotor can be described as

(7) $$\left\{\begin{array}{c} f_{ax}=-(k_{i}i_{ax}+k_{h}x_{a})-\frac{1}{2}\left(1+\frac{l_{r}}{l_{m}}\right)mw_{r}^{2}r_{d}\cos(w_{r}t+\varphi_{0})\\ f_{bx}=-(k_{i}i_{bx}+k_{h}x_{b})-\frac{1}{2}\left(1-\frac{l_{r}}{l_{m}}\right)mw_{r}^{2}r_{d}\cos(w_{r}t+\varphi_{0})\\ f_{ay}=-(k_{i}i_{ay}+k_{h}y_{a})-\frac{1}{2}\left(1+\frac{l_{r}}{l_{m}}\right)\left(mw_{r}^{2}r_{d}\sin(w_{r}t+\varphi_{0})-mg\right)\\ f_{by}=-(k_{i}i_{by}+k_{h}y_{b})-\frac{1}{2}\left(1-\frac{l_{r}}{l_{m}}\right)\left(mw_{r}^{2}r_{d}\sin(w_{r}t+\varphi_{0})-mg\right) \end{array}\right.$$

It can be seen from Equation (7) that the equations in the four directions are decoupled, which greatly simplifies the design of the force feedback controller. However, when the distance between mechanical bearings and magnetic bearings is large, or if a more accurate force compensation should be considered, *l_m_* cannot approximately be considered as *l_ab_*. At this time, multiple degrees of freedom in the radial direction of the system will be coupled with each other, so the system will be more complex. This complex situation will be further studied and will not be considered in this paper for the time being.

### 2.3. AMB Force Compensation System

**Figure 6:**
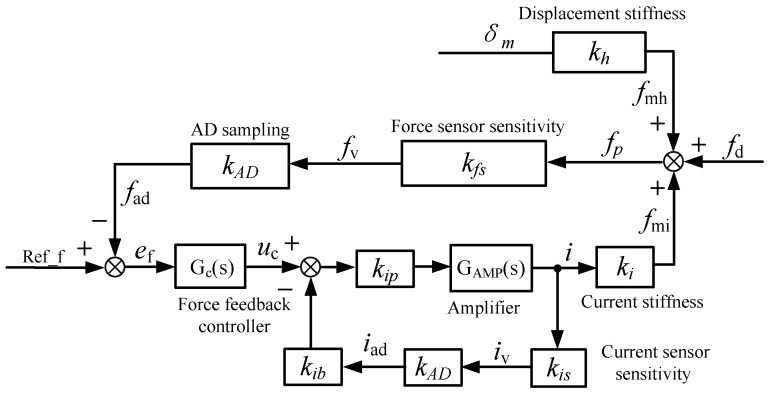
Block diagram of the AMB force compensation system.

After analyzing the force situation of the rotor, the transfer function model of the AMB force compensation system is going to be established next. Since the rotor is not suspended, the control system is much simpler than the magnetically suspended rotor system. According to Figure 5 and Equation (7), since each degree of freedom in the radial direction is independent, they can be analyzed separately. The block diagram of the AMB force compensation system is shown in Figure 6.

In Figure 6, *f_d_* is the external disturbing force, and it mainly refers to the gravity and unbalanced force in Equation (7); *f_mh_* and *f_mi_* are the displacement stiffness force and current stiffness force of the AMB, respectively; *f_p_* is the force value acting on the mechanical bearing, which has the same magnitude as the mechanical bearing force but in the opposite direction; *k_fs_* is the sensitivity of the force sensor; *k_AD_* is the amplification factor of AD sampling; Ref_f is the reference input, since the force acting on the mechanical bearing *f_p_* is expected to be zero, so Ref_f = 0. *G_c_(s)* is the transfer function of the force feedback controller; *G_AMP_(s)* is the transfer function of the amplifier link; *k_is_* is the sensitivity of the current sensor; *u_c_* is the output of the force feedback controller, and *i* is the control current of the magnetic bearing. The working principle of the AMB force compensation system is given in the description of Figure 5, and the system is introduced in detail in the following content.

**Figure 7:**
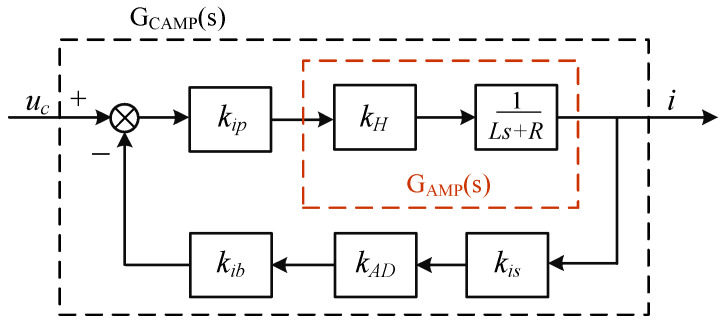
Block diagram of the AMP link considering current feedback.

The AMB force compensation system is not complex, and some parameters have been determined as the fixed parameters before designing the force feedback controller, such as *k_i_*, *k_h_*, *k_fs_*, and *k_AD_*. The values of these parameters are given in the Section 3. In addition, since the rotor is supported by mechanical bearings, the geometric centers of mechanical bearings and magnetic bearings are designed to be coincidental in this article. So, *δ_m_* is designed to be zero, and *f_mh_* = 0. It can be seen from Figure 6 and Equation (7) that the compensation of external disturbance force basically depends on the current stiffness force. The current stiffness force is controlled by the control current *i* of the magnetic bearing. In the hardware circuit, the control current *i* of the magnetic bearing coils is controlled by the equivalent voltage loaded on the magnetic bearing coil, and the equivalent voltage is controlled by the duty cycle of an H-bridge based on the controller output. In order to achieve high-frequency force compensation through the magnetic bearings, the AMP module must have sufficient bandwidth, so the current feedback loop is considered. The block diagram of the AMP link considering current feedback is shown in Figure 7.

The input of the AMP module is the controller output *u_c_*, and the output of the AMP module is the coil current *i*. The transfer function of the open-loop AMP module can be described as

(8) $$G_{AMP}(s)=\frac{k_{H}}{Ls+R}$$

where *k_H_* is the equivalent amplification factor of the H-bridge circuit; *L* is the inductance of the magnetic bearing coil and *R* is the resistance of the coil; *s* is the complex variable of the transfer function. Due to the narrow bandwidth of the open-loop amplifier link, the current feedback link is added to broaden its bandwidth. The closed-loop AMP model can be described as

(9) $$G_{CLAMP}(s)=\frac{k_{H}k_{ip}}{Ls+R+k_{H}k_{ip}k_{is}k_{ib}k_{AD}}$$

where *k_ip_* is the current amplification coefficient, *k_ib_* is the current feedback coefficient, and *k_is_* is the sensitivity of the current sensor. The bandwidth of the open-loop and closed-loop AMP model can be, respectively, described as

(10) $$\left\{\begin{array}{c} \omega_{AMP}=\frac{R}{L}\\ \omega_{CAMP}=\frac{R+k_{H}k_{ip}k_{is}k_{ib}k_{AD}}{L} \end{array}\right.$$

where *ω_AMP_* and *ω_CAMP_* are the bandwidths of the open-loop and closed-loop AMP link, respectively. Obviously, the current feedback link increases the bandwidth of the AMP link, and it can be adjusted by choosing different values of *k_ib_* and *k_ip_*. Since the maximum rotational speed of the standard device in this paper is designed at 8000 r/min, the bandwidth closed-loop AMP link is set to be greater than 150 Hz. The relevant parameters are listed in Table 1.

After the closed-loop AMP link is determined, the force feedback controller *G_c_(s)* is further designed and analyzed. Since the AMB force compensation system is not complex and it is designed first, a simple proportional integral (PI) controller is preferentially considered. More advanced algorithms will be tried in future research to improve the disturbance suppression performance of the AMB force compensation system.

When the force feedback controller is first designed as a *K_p_* controller

(11) $$G_{c}(s)=K_{p}$$

Then, the transfer function of the preceding channel of the closed-loop system is

(12) $$G\left(s\right)=\frac{K_{p}k_{H}k_{ip}k_{i}}{Ls+R+k_{H}k_{ip}k_{is}k_{ib}k_{AD}}$$

And the transfer function of the feedback channel is *H(s) = k_fs_k_AD_*. Since the reference input is a constant value, the system input is *R_in_(s)* = 1/*s*. The steady-state error of the system is

(13) $$E_{ss}=\underset{t\to \infty }{\lim}e_{f}(t)=\underset{s\to 0}{\lim}\frac{sR_{\mathrm{in}}(s)}{1+G(s)H(s)}=\frac{R+k_{H}k_{ip}k_{is}k_{ib}k_{AD}}{R+k_{H}k_{ip}k_{is}k_{ib}k_{AD}+K_{p}k_{H}k_{ip}k_{i}k_{fs}k_{AD}}$$

*E_ss_* is the steady-state error. Obviously, when the system is only controlled by a *K_p_* feedback controller, there is a steady-state error in the system. It means that the AMB force compensation system cannot accurately control the force acting on the coupling bearing.

When the force feedback controller is designed as a PI controller

(14) $$G_{c}=K_{p}+\frac{K_{i}}{s}$$

When the system input is *R_in_*(*s*) = 1/*s*, the steady-state error of the system is

(15) $$E_{ss}=\underset{t\to \infty }{\lim}e_{f}(t)=\underset{s\to 0}{\lim}\frac{sR_{\mathrm{in}}(s)}{1+G(s)H(s)}=\frac{s(R+k_{H}k_{ip}k_{is}k_{ib}k_{AD})}{(R+k_{H}k_{ip}k_{is}k_{ib}k_{AD})s+(K_{p}s+K_{i})k_{H}k_{ip}k_{i}k_{fs}k_{AD}}=0$$

Equation (15) shows that when the force feedback controller is designed as a PI controller, the steady-state error of the AMB force compensation system is zero. It means that the AMB force compensation system can theoretically control the static disturbing force acting on the coupling bearing to the defined reference input (normally zero).

## 3. Experimental Results

### 3.1. Experimental Platform

**Figure 8:**
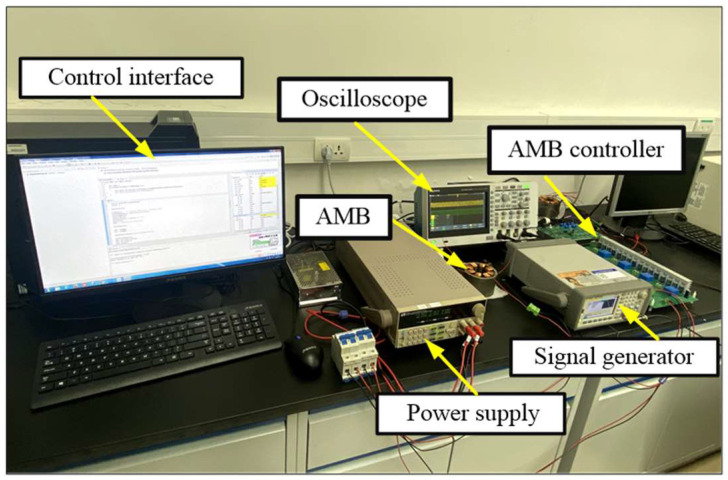
Semi-physical simulation experimental platform of the AMB force compensation system.

The disturbance suppression method based on AMB force compensation proposed in this paper is verified in the semi-physical simulation experimental platform, as shown in Figure 8. The experimental platform mainly includes the power supply (model: ITECH IT6332A), the AMB, the controller, the signal generator (model: Agilent 33500B), the oscilloscope, and the human–computer interaction interface. The core computing unit of the AMB control board is DSP TMS320F28335, and the control cycle of the AMB force compensation control system is 150 us. The human–computer interface is the debugging interface of Code Composer Studio 6.1. In addition, the H-bridge power supply voltage in the power amplifier board is 28 V, and the modulation frequency of the PWM wave is 20 kHz. The signal generator is used to simulate the external disturbance force, which will be conditioned by the conditioning circuit in the control board and collected by the AD module of the DSP.

It should be noted that the state and calculation of the force acting on the mechanical bearing and the state of AMB force are numerically simulated by defining variables in the DSP program. The current sensor and H-bridge circuit are located in the power amplifier circuit board of the AMB controller, and, together with the magnetic bearing coil, they constitute the AMP link, as shown in in Figure 6. The current stiffness force can be calculated by the coil current of the magnetic bearing, and it is also numerically simulated by the DSP operation link. In addition, the parameters of the AMB force compensation system are shown in Table 1.

### 3.2. Start–Up Experiments and Step Disturbance Suppression Performance

In order to verify the anti-interference ability and dynamic performance of the AMB force compensation system, two groups of experiments are first carried out in this subsection. The first group’s experiments are the start-up experiments of the AMB system when the mechanical bearing is subjected to a constant external disturbance force, such as the gravity of the rotor. These experiments also correspond to the actual situation when the AMB system is started. The second group’s experiments are the step disturbance force suppression experiments. The AMB force compensation system is first enabled, then a step disturbance force is added, and the force response of the system is observed. The first group’s experiments’ results are shown in c.

**Figure 9:**
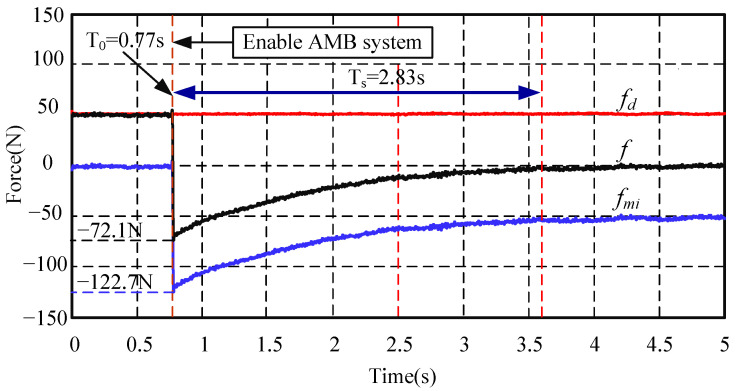
Start-up experiments of the AMB force compensation system when the mechanical bearing is subjected to a constant external disturbance force. Note: high frequency interference signal has been filtered out.

In Figure 9, the red line is a constant external disturbance force of 50 N; the blue line is the compensation force provided by the AMB force compensation system; the black line is the force acting on the mechanical bearing. At the initial time, the AMB force compensation system is not activated. All of the constant external disturbance force is applied to the mechanical bearing, so the mechanical bearing bears a force of 50 N. Then, the AMB force compensation system is enabled at 0.77 s. At this time, the AMB force compensation system rapidly provides a compensation force of 122.7 N at 0.086 s. The force acting on the mechanical bearing rapidly changed from 50 N to −72.1 N, and then reached a stable state at 2.83 s. It can be seen from the experimental results that the mechanical bearing will experience an impact force, but it can be reduced by adjusting the integral coefficient, and accordingly, the steady-state time will be extended.

**Figure 10:**
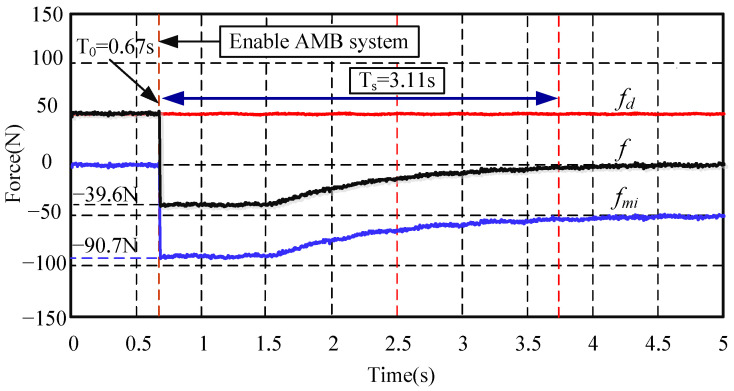
Start-up experiments of the AMB system considering control current saturation. Note: high frequency interference signal has been filtered out.

Since the semi-physical simulation experiment is adopted in this paper, the saturation of AMB force is not considered in the start-up experiments. In fact, if the bias current is 0.6 A and the current stiffness is 150 N/A, the maximum output force of the AMB is 90 N. So, the coil current is further limited in the algorithm, and the start-up experiment is carried out again. The experimental result is shown in Figure 10.

It can be seen from Figure 10 that, when the AMB force compensation system is activated at 0.67 s, the AMB immediately provides a compensation force of −90.7 N. The AMB is in a current saturation state. The AMB system reaches a steady state at 3.11 s. The experimental results show that the system is still stable and can eliminate the influence of static disturbance.

**Figure 11:**
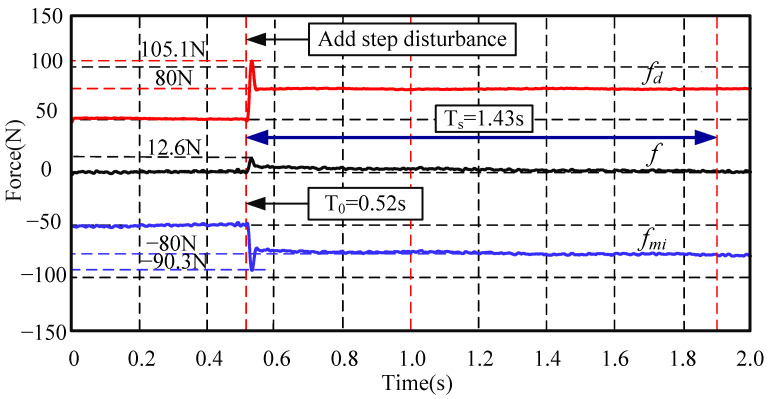
AMB compensation performance when the closed-loop system encounters a step disturbance force.

Then, when the system shown in Figure 10 becomes stable, the second group of experiments, the step disturbance suppression experiments, is carried out. The experimental results are shown in Figure 11.

In Figure 11, the AMB force compensation system has been activated from the initial time. At the initial time, although the rotor is subject to constant external disturbance, due to the AMB force compensation system, the force acting on the mechanical bearing is almost zero. At 0.515 s, an additional step disturbance force of 30 N is suddenly applied to the rotor, which is simulated by the signal generator. Since the signal generator itself would produce an overshoot, a maximum force of 55.1 N is actually applied to the system, and the total external disturbance force could reach 105.1 N when considering the initial constant force. At this time, it can be seen that the AMB force compensation system rapidly increases a force of −40.3 N, and the total AMB force could reach −90.3 N when considering the initial compensation force. The peak value of step disturbance is suppressed by at least 88%. The system reaches a steady state at 1.43 s. Due to the large values of *K_P_* and *K_i_*, the AMB force compensation system shows a fast response speed. The experimental results show that the AMB force compensation system has a good ability to suppress step disturbance.

### 3.3. Sinusoidal Disturbance Suppression Performance

**Figure 12:**
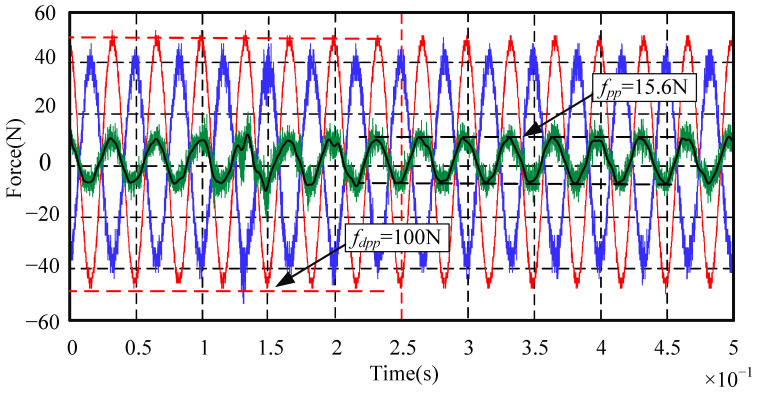
Sinusoidal disturbance suppression experiment when the disturbance frequency is 30 Hz.

**Figure 13:**
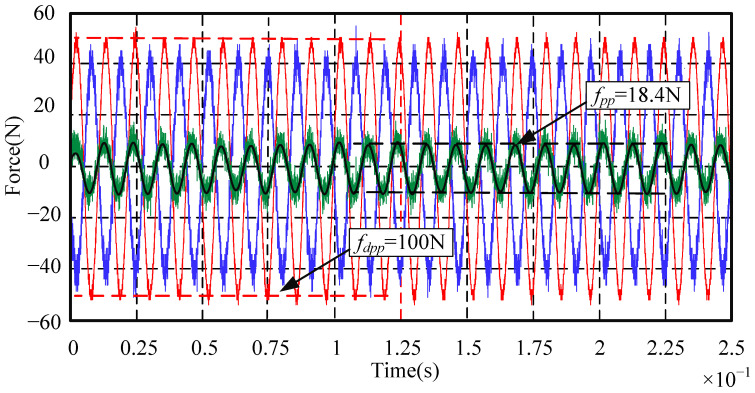
Sinusoidal disturbance suppression experiment when the disturbance frequency is 90 Hz.

**Figure 14:**
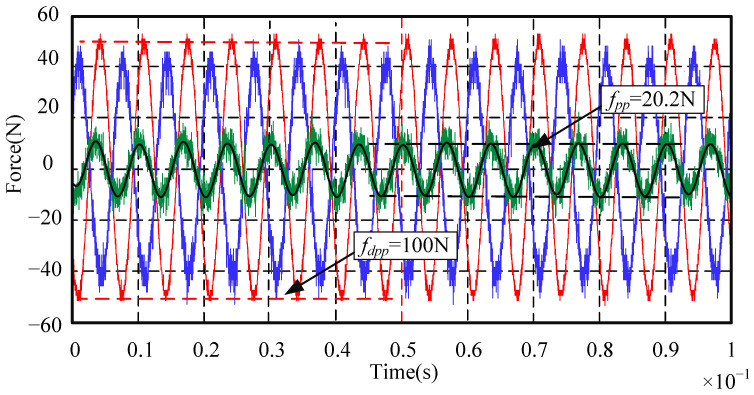
Sinusoidal disturbance suppression experiment when the disturbance frequency is 150 Hz.

When the rotor reaches a high rotational speed, the main disturbance force is the synchronous frequency disturbance force related to the rotation frequency. The suppression effects of the synchronous frequency disturbance force are emphatically analyzed. Since the maximum rotational speed of the standard device in this paper is designed as 8000 r/min, the highest frequency of the synchronous frequency disturbance force is set to 150 Hz. In order to observe the synchronous frequency disturbance suppression effect in the whole rotational speed range, the signal generator is used to simulate the sinusoidal disturbance force with a peak-to-peak value of 100 N and with frequencies of 30 Hz, 90 Hz, and 150 Hz, respectively. The disturbance suppression performance of the AMB force compensation system is shown in Figure 12, Figure 13 and Figure 14.

In Figure 12, Figure 13 and Figure 14, the red line is the sinusoidal disturbance force; the blue line is the compensation force provided by the AMB force compensation system; the green line is the force acting on the mechanical bearing; the black line is the filtered green line after filtering out the high-frequency noise for the convenience of analysis and observation. It can be seen from these three figures that, when the frequency of the external disturbance force is 30 Hz, 90 Hz, and 150 Hz, respectively, the peak-to-peak values of the force acting on the mechanical bearing after adding the AMB force compensation system are reduced to 15.6 N, 18.4 N, and 20.2 N, respectively. It means that the external disturbance forces are suppressed by 84.4%, 81.6%, and 79.8%, respectively. The experimental results show that the AMB force compensation system can significantly suppress the external disturbance force. In addition, the performance of the AMB force compensation system can be better when the parameters of the controller are further optimized.

## 4. Discussion

In the process of establishing the AMB force compensation system, a short distance between the mechanical bearing and the AMB is regarded, so *l_ab_* and *l_m_* are approximately equal. And the whole system is simplified into four systems with independent degrees of freedom. However, in some practical applications, there may be a large distance between the mechanical bearing and the AMBs. In addition, in order to suppress external disturbance more precisely, *l_ab_* and *l_m_* are considered approximately not equal. In this case, the four degrees of freedom of the radial AMB force compensation system are coupled, so it is necessary to consider the four degrees of freedom at the same time when designing the controller of the AMB force compensation system, which will increase the difficulty of the controller design. This is also our future research direction.

## 5. Conclusions

In this paper, focusing on the problem of disturbance force suppression of the rotational speed standard device, a force compensation control strategy based on the AMB force compensation system is proposed. First, the basic structure of the AMB force compensation system is designed. Then, the force model of the AMB force compensation system and the single degree of freedom AMB force compensation control system is analyzed and established. Finally, a semi-physical simulation platform is established to verify the start-up performance, step disturbance suppression performance, and unbalanced disturbance suppression performance of the AMB force compensation system. The experimental results show that the peak value of step disturbance is suppressed by at least 88%, and the external unbalanced sinusoidal disturbance forces can be suppressed by about 84.4%, 81.6%, and 79.8% at frequencies of 30 Hz, 90 Hz, and 150 Hz, respectively.

## Figures and Tables

**Table 1:** Parameters of the AMB force compensation system.

Symbol	Parameter	Value
*k_AD_*	Sampling amplification factor	4096/3
*R*	Resistance of magnetic bearing coil	1.3 Ω
*L*	Inductance of magnetic bearing coil	5.19 mH
*k_i_*	Current stiffness of magnetic bearing	150 N/A
*k_h_*	Displacement stiffness of magnetic bearing	192.4 N/mm
*k_is_*	Sensitivity coefficient of current sensor	0.5 V/A
*k_fs_*	Sensitivity of force sensor	0.01 V/N
*K_p_*	Proportional of PI controller	11
*K_i_*	Integral coefficients of PID controller	15
*k_ib_*	Feedback coefficients of the current loop	0.1
*k_ip_*	Proportional coefficients of the current loop	5

## Data Availability

The data presented in this study are available on request from the corresponding author.
